# Exosome-mediated Hic-5 regulates proliferation and apoptosis of osteosarcoma via Wnt/β-catenin signal pathway

**DOI:** 10.18632/aging.103546

**Published:** 2020-12-13

**Authors:** Liansheng Sha, Deying Ma, Cuili Chen

**Affiliations:** 1 Department of Orthopedics, People’s Hospital of Rizhao, Rizhao 276826, Shandong Province, China

**Keywords:** osteosarcoma, Hic-5, exosome, Wnt/β-catenin

## Abstract

The expression of Hic-5 was detected in osteosarcoma patients and osteosarcoma cell lines by RT-PCR. Then RFP-sh-Hic-5 was transfected into osteosarcoma cell lines. The effect of Hic-5 on cell viability, proliferation and apoptosis were assessed by MTT, EdU kit and Flow cytometry. The exosomes were isolated from MG-63 cell supernatant by an Exosome Isolation Kit. The exosome-Hic-5 was confirmed by transmission electron microscope, particle size detection and RT-PCR. Next, exosome-Hic-5 treated cells were explored the cell viability, proliferation and apoptosis. Further, Co-IP assay was employed for identifying the relationship between Hic-5 and smad4. TCF/LEF and the protein level of components of wnt/β-catenin signals were detected by TOP luciferase assay and western blot. Hic-5 was upregulated in osteosarcoma tissues and cell. Forced decreased expression Hic-5 inhibited the proliferation of osteosarcoma cell lines, and induced apoptosis of MG-63 and HOS. *In vivo*, silencing Hic-5 remitted the tumor progression. Further, we isolated the exosomes from MG-63 supernatant, exosomes concluding Hic-5 would regulated the proliferation and apoptosis level of MG-63 and HOS cells. Further, Hic-5 interacted with smad4 and regulated Wnt/β-catenin signal by decreasing TCF/LEF activity. Silencing Hic-5 inhibited the proliferation and induced apoptosis of osteosarcoma cell via inactivating Wnt/β-catenin signal by exosome pathway.

## INTRODUCTION

Osteosarcoma (OS) is the most common primary malignant tumor of bone, which originates from mesenchymal stem cells. The most common metastatic sites of osteosarcoma are the lungs (more than 85%) and bones [1, 2]. Before the introduction of chemotherapy, the prognosis of osteosarcoma was poor, and the survival rate of patients with osteosarcoma was less than 20% [1]. At present, the treatment of newly diagnosed osteosarcoma includes preoperative chemotherapy, surgery and postoperative chemotherapy [3]. Neoadjuvant chemotherapy with methotrexate, doxorubicin, cisplatin and cyclophosphamide has increased the survival rate of osteosarcoma to 60% to 70% [4]. Therefore, it is imperative to explore new treatment methods and strategies.

An exosome is a vesicle with lipid bilayer membrane structure secreted by cells under physiological or pathological conditions, which contains proteins, double-stranded DNA (dsDNA), RNA and other components [5, 6]. The exosome can transmit biological signals to the receptor cells, resulting in functional changes of the corresponding cells. In recent years, a number of studies have shown that exosome bodies are closely related to the genesis, migration, drug resistance, angiogenesis and immune regulation of malignant tumors [7–9]. It was reported that the combination of exosome derived from bone marrow mesenchymal stem cells and doxorubicin can hinder the development of osteosarcoma [10]. Therefore, an in-depth study of the effect of exosome on the development of osteosarcoma can provide a theoretical basis for the clinical treatment of osteosarcoma.

Hic-5 (hydrogen peroxide inducible clone 5, also known as TGFB1l1), which was first isolated and cloned from mouse osteoblast MC3T3 induced by TGF-β, can also be expressed under the induction of hydrogen peroxide [11, 12]. Hic-5 was expressed in adherent plaques or nuclei, and is mainly limited to adherent plaques. Hic-5 acts as a regulatory molecule to interact with a variety of proteins to form an adhesion scaffold for integrin and other structural and signal molecular responses, which regulates the assembly of integrins [13]. In the nucleus, Hic-5, as a coregulator, selectively interacts with nuclear receptors and target gene promoters, and promotes or inhibits the expression of target genes [14]. Hic-5 can be used as a junction protein to recruit co-initiators and bind to nuclear receptors to form nuclear receptor response modules. Hic-5 is a signal-specific cofactor of cellular TGF-β1. Its LIM domain binds to the MH2 domain of the regulatory protein Smad3 of TGF-β1 and blocks the transcriptional response mediated by Smad3 protein [15]. Hic-5 is an important target gene of pathogenic myofibroblast-like cells in fibrotic diseases. Hic-5 is involved in the regulation of TGF- β1-induced proliferation and differentiation of fibroblasts into myofibroblasts. The cellular process is an important new target gene for the occurrence of fibrotic diseases [16].

Wnt is a kind of secretory glycoprotein, which acts through autocrine or paracrine pathway. β-catenin (β-catenin) is an important signal transduction factor in Wnt signaling pathway and is a multifunctional protein [17]. Free β-catenin can enter the nucleus and regulate gene expression. In addition, β-catenin can bind cadherin to form an adhesive band at the cell junction of the body. Wnt signal pathway can promote the continuous accumulation of intracellular β-catenin, and the abnormal expression of β-catenin can induce tumorigenesis [18]. Wnt/β-catenin can accelerate tumor cell infiltration and distant metastasis, thus accelerating tumor progression. It has been found that there is abnormal expression of Wnt/β-catenin in canceration of multiple organs and tissues, such as esophageal cancer, colorectal cancer, renal cell carcinoma, gastric cancer, liver cancer and breast cancer [19].

In this study, we described the role of Hic-5 in osteosarcoma cells. Silencing Hic-5 can inhibit proliferation and promote apoptosis of osteosarcoma cells. It is further found that Hic-5 exists in the exosome of osteosarcoma cells. Exosome-derived Hic-5 affects the development of osteosarcoma through Wnt/β-catenin pathway.

## RESULTS

### Hic-5 is up-regulated in osteosarcoma tissues and cells

**Figure 1:**
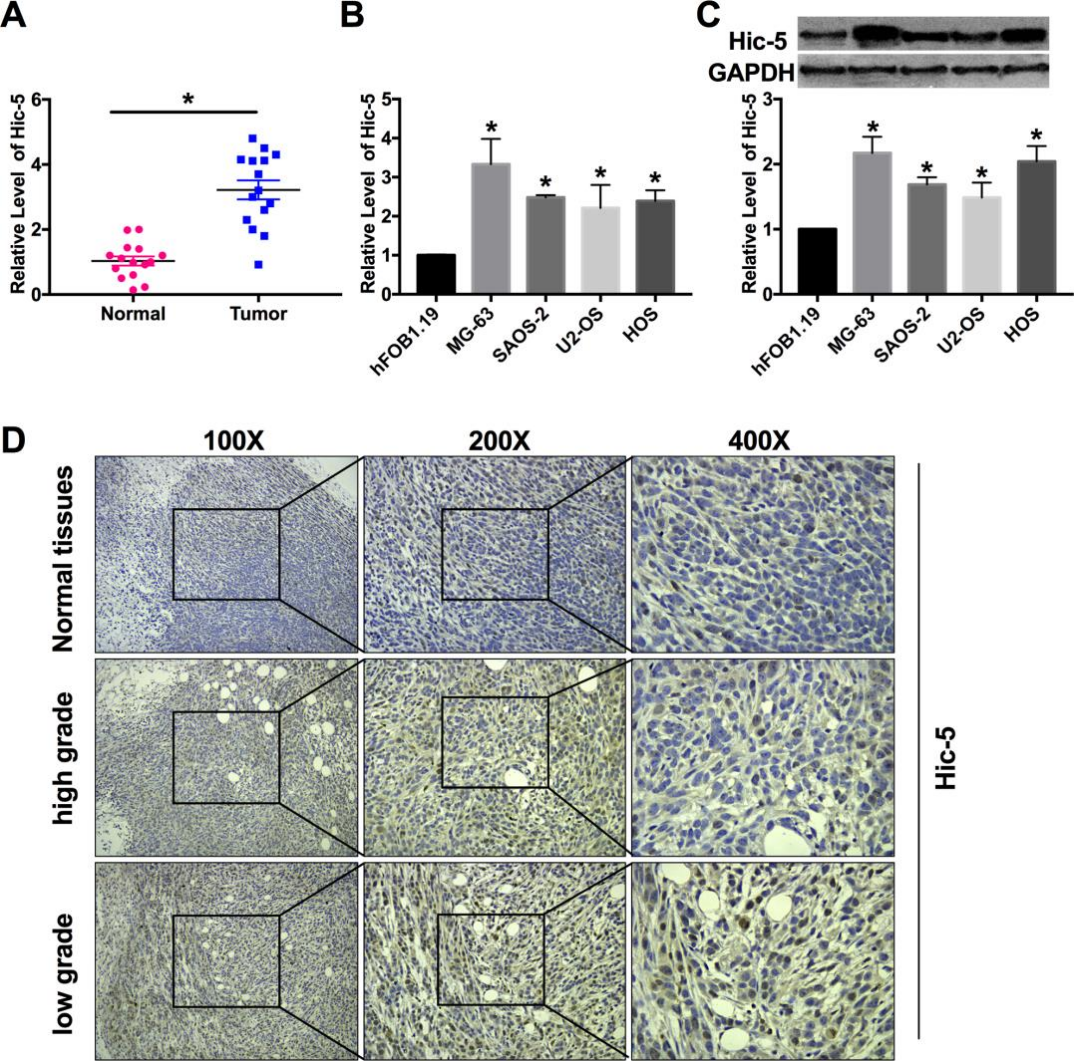
**Hic-5 was up-regulation in human osteosarcoma tissues and cell lines.** (**A**) RT-PCR was employed to detect the expression of Hic-5 in tumor and normal tissues. n=15, **P<0.01. (**B** and **C**) The mRNA and protein expression of Hic-5 was measured in MG-63, HOS, U2-OS and SAOS-2 cells, hFOB1.19 cells were described as control. n=8, *P<0.05. (**D**) Hic-5 staining in slides from osteosarcoma (high grade and low grade) and adjacent normal bone tissues.

RT-qPCR was performed to detect Hic-5 expression in osteosarcoma tissues and cell lines. The results showed miR-496 expression was up-regulated in the OS tissues than that in the normal group (Figure 1A). Compared with hFOB1.19 cells, the expression level of Hic-5 was significantly increased in MG-63, HOS, U2-OS and SAOS-2 cells (Figure 1B). The protein level of Hic-5 was measured by western blot, which was upregulation in four OS cell lines (Figure 1C). In adjacent normal bone tissue biopsies (53.3%, 8/15), only a low level of Hic-5 was present. More frequency (46.7%, 7/15) of higher expressed Hic-5 was present in low grade osteosarcoma tissues. In contrast with low grade osteosarcoma tissues, a decreased staining for Hic-5 was observed in most high grade osteosarcoma patient tissues (53.3%, 8/15) (Figure 1D).

### Knockdown of Hic-5 prevents growth and induces apoptosis of OS

**Figure 2:**
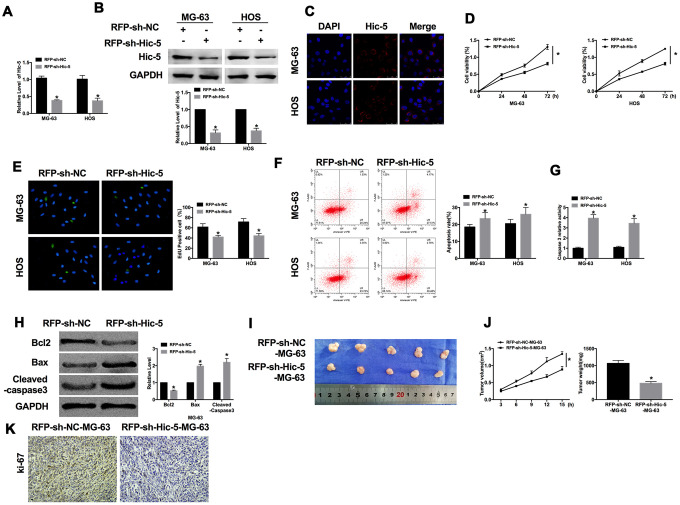
**Dysfunction of Hic-5 regulated the proliferation and apoptosis in osteosarcoma.** The transfection efficiency was verified by RT-PCR (**A**, n=6) western blot (**B**, n=3), and immunofluorescence (**C**, n=6). *P<0.05. (**D**) MTT assay was performed to detected cell viability in OS cells. n=10, *P<0.05. (**E**) The proliferation ability of OS cells was measured by Edu assay. (green, Edu^+^, blue, DAPI). n=6, *P<0.05. (**F**) The apoptosis rate was detected by flow cytometry. n=5, *P<0.05. (**G**) The caspase3 activity was assessed in OS cells. n=6, *P<0.05. (**H**) The apoptosis-associated protein (Bcl2, Bax and Cleaved-caspase3) level was detected by western blot. n=6, *P<0.05. (**I**) Effects of Hic-5 knockdown on the size of MG-63 xenograft tumors in nude mice. (**J**) The tumor volume and weights of removed tumor were calculated. n=10, *P<0.05. (**K**) Representative Ki-67 staining of tumor sections in different group.

For further explore the role of Hic-5, we created red fluorescence plasmid (RFP)-shRNA for knockdown Hic-5 (RFP-sh-Hic-5), RFP-sh-NC was described as an negative control. RT-PCR and western blot were performed to detect the transfection efficiency (Figure 2A and 2B), and immunofluorescence experiment also verified the RFP-shRNA functional entered the OS cells (Figure 2C). Cell viability was evaluated in MG-63 and HOS cells, as Figure 2D shown, downregulation of Hic-5 inhibited the cell viability of OS cells. We observed that knockdown of Hic-5 prevented the proliferation of OS cells, which was indicated by Edu assay (Figure 2E). Then we investigated the effect of Hic-5 on apoptosis of OS cells. Flow cytometry was employed to detect the apoptosis rate of MG-63 and HOS cells after RFP-shRNA transfection. RFP-sh-Hic-5 dramatically increases the number of apoptosis cell (Figure 2F). Compared with RFP-sh-NC, the caspase3 activity was increasing in RFP-sh-Hic-5 group, which was measured by caspase3 activity kit (Figure 2G). Meanwhile, we detected the expression of apoptosis-relative protein, Bcl2, Bax and Cleaved-caspase3. The results revealed the downregulation of Bcl2 and upregulation of Bax, Cleaved-caspase3 (Figure 2H).

Subsequently, we constructed a stable MG-63 cell line with low expression of Hic-5 for *in vivo* tumor experiment. MG-63 cells were subcutaneously injected in right lower limb of the nude mice. Tumor size was measured every 3 days. After another 15 d of injection, the tumors were removed (Figure 2I). Tumor volume and weight were calculated, the tumors from RFP-sh-Hic-5-MG-63 cells injection mice were smaller and lighter than tumor from RFP-sh-NC-MG-63 cells injection mice (Figure 2J). Further, immunohistochemical analysis of Ki-67 in two group samples performed that knockdown of Hic-5 inhibited the tumor progression (Figure 2K). Taken together, knockdown of Hic-5 would inhibit the proliferation and promote apoptosis of OS.

### Extraction, purification and confirmation of exosomes

**Figure 3:**
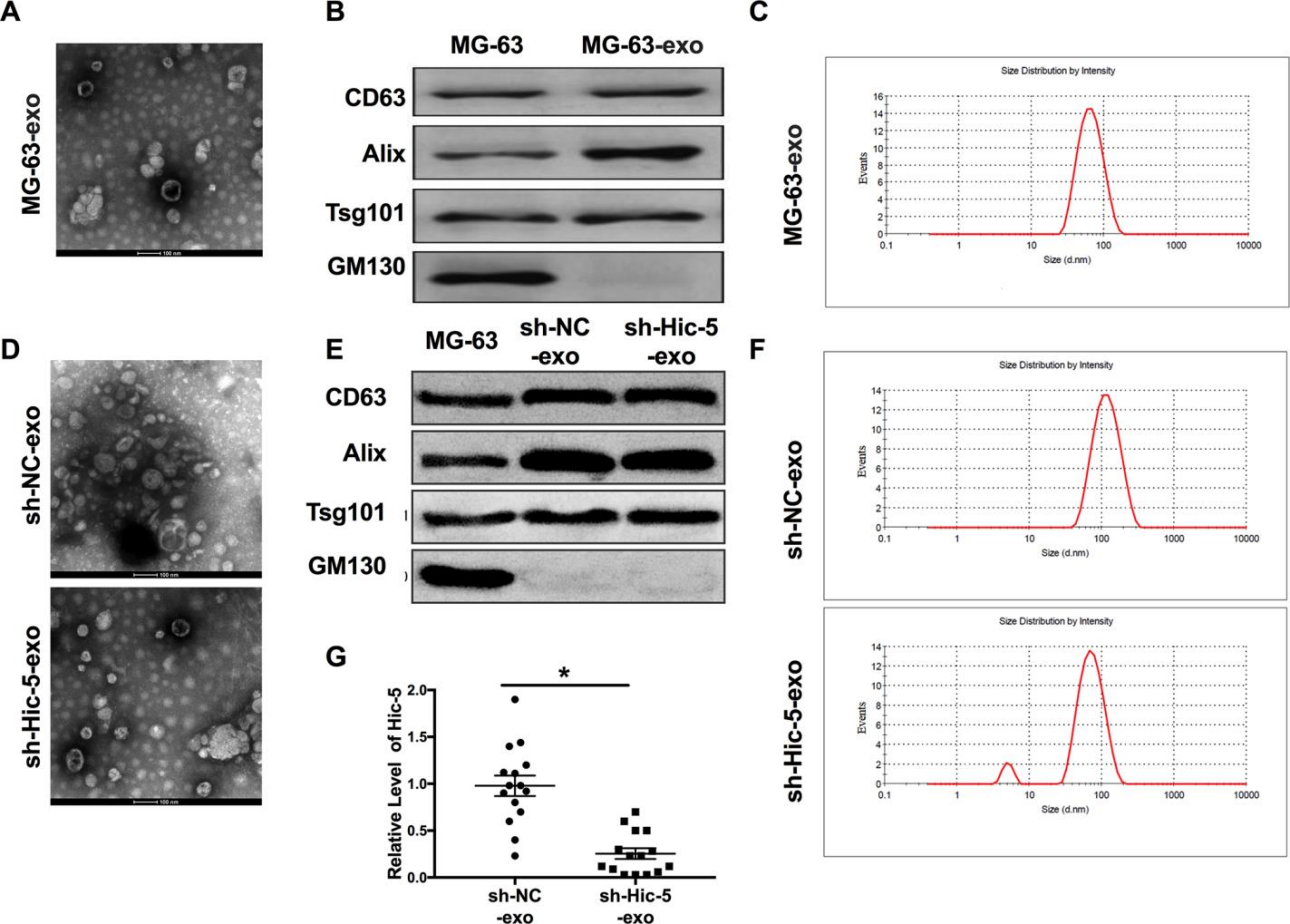
**Exosomes derived from OS supernatant were identified.** (**A**) The morphology of exosome was observed by TEM. (**B**) The biomarker (CD63, Alix, Tsg101 and GM130) of exosome were detected by western blot. (**C**) Exosome particle size (about 30-150 nm) were analyzed. The characteristics of sh-Hic-5-exo and sh-NC-exo were confirmed by TEM (**D**), biomarker (**E**) and particle size (**F**). (**G**) The mRNA expression of Hic-5 was measured in sh-Hic-5-exo and sh-NC-exo. n=4, *P<0.05.

Exosome-specific membrane structures and contents are widely involved in material exchange and information exchange between osteosarcoma cells, such as regulating the microenvironment of osteosarcoma, regulating the expression of Wnt/β-catenin signal and TGF-β signal pathway, inducing tumor cell immune escape and so on. The exosome body of osteosarcoma can also cooperate with antigen presenting cells to activate the immune response of the body and play an anti-tumor role. We speculated that Hic-5 may also affect the development of OS in the form of exosomes. Then we isolated the exosomes from MG-63 supernatant. Exosomes were identified by transmission electron microscopy (TEM, Figure 3A), biomarkers (CD63, Alix, Tsg101 and GM130, Figure 3B) and particle size (about 30-150 nm, Figure 3C) analysis. Then we extracted exosomes from MG-63 cells transfection with RFP-shRNA. Then the exosomes from sh-Hic-5-MG-63 (sh-Hic-5-exo) and RFP-sh-NC-MG-63 (sh-NC-exo) cells supernatant were identified by TEM (Figure 3D), biomarkers (Figure 3E) and particle size (Figure 3F). RT-PCR assay was performed to detect the expression level of Hic-5 from sh-Hic-5-exo and sh-NC-exo. Hic-5 was decreased in sh-Hic-5-exo (Figure 3G).

### Exosomal-Hic-5 regulates the proliferation and apoptosis of OS

**Figure 4:**
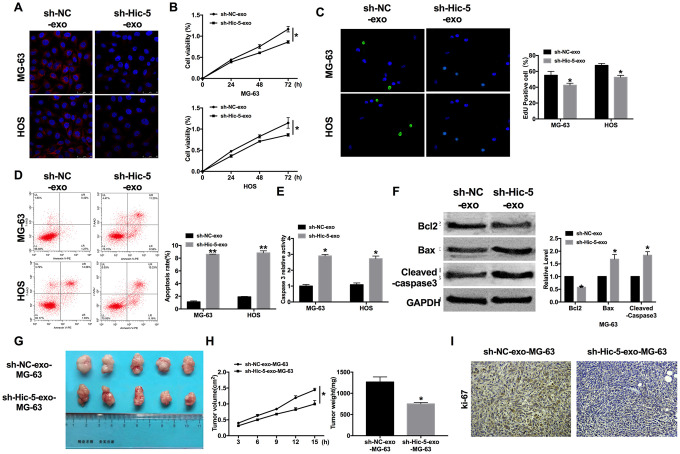
**Exosomal-Hic-5 effected the proliferation and apoptosis of OS.** (**A**) Immunofluorescence assays was performed to detect co-cultured results in OS cells. (**B**) MTT assay was performed to detected cell viability in co-cultured OS cells. n=10, *P<0.05. (**C**) The proliferation ability of co-cultured OS cells was measured by Edu assay. (green, Edu^+^, blue, DAPI). n=6, *P<0.05. (**D**) The apoptosis rate was detected by flow cytometry. n=5, *P<0.05. (**E**) The caspase3 activity was assessed in co-cultured OS cells. n=6, *P<0.05. (**F**) The apoptosis-associated protein (Bcl2, Bax and Cleaved-caspase3) level was detected by western blot. n=6, *P<0.05. (**G**) The size of exosomes co-cultured MG-63 xenograft tumors in nude mice. (**H**) The tumor volume and weights of removed tumor were calculated. n=15, *P<0.05. (**I**) Representative Ki-67 staining of tumor sections in different group.

For further explore the function of exosomal-Hic-5 in OS, we co-cultured sh-Hic-5-exo/sh-NC-exo with MG-63 and HOS cells. Then immunofluorescence assay was performed to detect Hic-5. Hic-5 was decreased in co-cultured with sh-Hic-5-exo cells (Figure 4A), the results revealed exosomal-Hic-5 could enter into OS cells. sh-Hic-5-exo also reduced cell viability in OS cells (Figure 4B), and inhibited proliferation of cells (Figure 4C). Apoptosis level was measured by flow cytometry. sh-Hic-5-exo promotes apoptosis rate of OS cells (Figure 4D), and increased caspase3 activity (Figure 4E). The down-regulated of Bcl2 and up-regulated of Bax, Cleaved-caspase3 once indicated that sh-Hic-5-exo could elevate apoptosis level (Figure 4F).

Then the MG-63 cells were collected after co-cultured with sh-Hic-5-exo/sh-NC-exo. The collected cells were subcutaneously injected in right lower limb of the nude mice. Tumor size was measured after another 15 d of injection, the tumors were removed (Figure 4G). Tumor volume and weight were calculated, the tumors from sh-Hic-5-exo-MG-63 cells injection mice were smaller and lighter than tumor from sh-NC-exo-MG-63 cells injection mice (Figure 4H). Further, immunohistochemical analysis of Ki-67 in two group samples performed that sh-Hic-5-exo-MG-63 cells injection mice showed poor ability of proliferation (Figure 4I). Taken together, knockdown of Hic-5 would inhibit the proliferation and promote apoptosis of OS via exosome pathway.

### Exosomal-Hic-5 regulates OS through Wnt/β-catenin signal pathway

**Figure 5:**
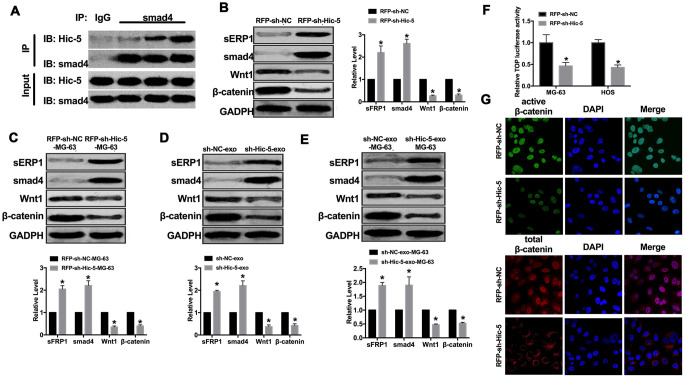
**Exosomal-Hic-5 regulated the development of OS via inhibiting Wnt/β-catenin signal pathway.** (**A**) CoIP assay was performed to confirm the relationship between Hic-5 and smad4. (**B**) The expression of Wnt/β-catenin signal pathway elements (sFRP1, Smad4, Wnt1and β-catenin) were detected in MG-63 cells after RFP-shRNA transfection. n=6, *P<0.05. (**C**) The expression of sFRP1, Smad4, Wnt1, β-catenin were detected in tumor from treated-MG-63 cell injection mice. n=10, *P<0.05. (**D**) The expression of Wnt/β-catenin signal pathway elements (sFRP1, Smad4, Wnt1and β-catenin) were detected in MG-63 cells after exosomes co-cultured. n=6, *P<0.05. (**E**) The expression of sFRP1, Smad4, Wnt1, β-catenin were detected in tumor from exosomes co-cultured-MG-63 cell injection mice. n=10, *P<0.05. (**F**) Wnt pathway activation by treatment with RFP-sh-Hic5/RFP-sh-NC regulates ß-Catenin signaling mediated TCF/LEF reporter activity. n=3, *P<0.05. (**G**) Subcellular localization of active β-catenin and total β-catenin in MG-63 cells by immunofluorescence staining.

Wnt pathway is the key pathway for cell development and growth regulation. Wnt/β-catenin pathway is the most classical Wnt signal pathway, and its abnormal activation plays an important role in the formation of malignant tumors. CoIP assay was carried out for detect the relationship between Hic-5 and Wnt/β-catenin signal pathway. We observed that Hic-5 could interact with smad4 in OS cells. Downregulation of sFRP1 and Smad4 could activate Wnt/β-catenin signaling pathway, thereby promoting tumor cell invasion and metastasis. Then we detected the expression of pathway elements (sFRP1, Smad4, Wnt1and β-catenin), upregulation of sFRP1, Smad4 and downregulation of Wnt1, β-catenin was performed in MG-63 cells transfection with RFP-sh-Hic-5 (Figure 5A and 5B). Similar results were achieved *in vivo* mice injection with RFP-sh-Hic-5-MG-63 cells (Figure 5C). Subsequently, we detected the expression of sFRP1, Smad4, Wnt1and β-catenin in MG-63 cells co-cultured with sh-Hic-5-exo/sh-NC-exo. Increased level of sFRP1, Smad4 and decreased level of Wnt1, β-catenin was performed in MG-63 cells co-cultured with sh-Hic-5-exo (Figure 5D). The mice injection with MG-63 cells co-cultured with sh-Hic-5-exo were showed consistent results (Figure 5E). Further, Knockdown of Hic-5 decreased the TOP luciferase activity which indicated the inhibition of Wnt/β-catenin signal activation (Figure 5F). Therefore, we found that protein expression of active β-catenin, but not total β-catenin, was reduced in RFP-sh-Hic-5-transfected MG-63 cells by immunofluorescence (Figure 5G).

## DISCUSSION

In our study, we found that Hic-5 was up-regulated in OS. Knockdown of Hic-5 prevented proliferation and promoted apoptosis of OS cells. Subsequently, we found that Hic-5 affected the development of osteosarcoma cells via regulating Wnt/β-catenin signal pathway through the exosome pathway.

Hic-5 has been proposed as a target molecule of the novel anti-cancer reagent. Omoto T et al. [20] revealed that Hic-5 promoted the expression of lysyl oxidase and collagen I in human control counterpart fibroblasts. Du X et al. [21] identified that stromal HIC-5 was a predictive risk factor for lymph node metastasis in human esophageal squamous cell carcinoma (ESCC) and that cancer-associated fibroblasts (CAFs)-derived Hic-5 regulated ESCC cell migration and invasion by regulating cytokines and modifying the extracellular matrix (ECM). Goreczny GJ also found that Hic-5 as a crucial regulator of ECM remodeling in CAFs by promoting fibrillar adhesion formation through a novel interaction with tensin1. Here, we found that Hic-5 played a key role in osteosarcoma progression by regulating the proliferation and apoptosis state. Further research, we would explore whether Hic-5 affects tumor development through fibroblasts.

Because of their biological activity, tumor-derived exosomes entered the field of vision of scientists. Researchers found that tumor cells can secrete more exosome bodies than normal cells. The contents of tumor-derived exosomes are highly variable and dependent on cell sources, but regardless of their origin, tumor-derived exosomes have a high degree of variability for recipient cells [22]. Because of its strong function, its ability to regulate the metastasis of tumor cells is mainly manifested in three aspects: first, tumor cells directly induce tumor metastasis by secreting exosome bodies. Second, tumor exosomes use miRNA as a vector to regulate the microenvironment of tumor cell growth [23]. and last tumor exosomes indirectly promote tumor metastasis by transforming distant stromal cells. In our study, we found that Hic-5 could regulate proliferation and apoptosis in osteosarcoma cells by exosome way.

Studies have shown that defects in Wnt/β-catenin signaling pathway can lead to a variety of developmental defects, and even lead to rheumatoid arthritis, cardiovascular disease and cancer [24]. Among them, the relationship between Wnt/β-catenin signaling pathway and cancer has become a research hotspot in recent years. It is reported that the members of Wnt/β-catenin signal pathway itself are oncogenes and tumor suppressor genes. Mutations of these genes and pathway components such as APC, β-catenin, Axin and so on will lead to inappropriate activation of the pathway, which is closely related to tumorigenesis. It has been found that the occurrence of colorectal cancer is related to the uncontrolled accumulation of intracellular β-catenin and the mutation of APC. Wnt2 is overexpressed in many stages of the development of colorectal cancer [25], and Wnt5a is overexpressed in breast cancer, colorectal cancer, lung cancer, prostate cancer and melanoma [26, 27]. In recent years, the relationship between Wnt/β-catenin signaling pathway and human diseases, especially tumors, has been gradually revealed, which provides a new way to prevent and control diseases. Wnt/ β-catenin signal pathway is also closely related to osteosarcoma [28]. In the Wnt/β-catenin signal pathway, β-catenin may play a leading role in the biological characteristics of osteosarcoma [29]. Some studies have shown that the level of β-catenin in osteosarcoma is much higher than that in normal tissue [30]. The levels of Wnt3a, β-catenin and LEF1 in human osteosarcoma cells were significantly higher than those in embryonic osteoblasts. Vijayakumar et al. [31] found that 50% of human sarcoma tissues and 65% of sarcoma cell lines showed an up-regulation of Wnt/ β-catenin signaling pathway. The high expression of β-catenin and some target genes can promote the development and metastasis of osteosarcoma, especially lung metastasis. Iwaya et al. [32] found that osteosarcoma cells with high lung metastatic potential have strong expression of β-catenin in cytoplasm and nucleus, indicating that the high expression of β-catenin can be used as a biomarker for detecting lung metastatic potential of osteosarcoma. Rubin et al. [33] established a 143B osteosarcoma cell line with overexpression of Wnt inhibitor-1 (WIF-1). After subcutaneous injection of this cell line into nude mice, the tumor growth rate *in situ* and lung metastasis rate decreased significantly. They also found a decrease in WIF-1 expression in 76% of osteosarcomas. These results suggest that WIF-1 can delay the development and metastasis of osteosarcoma by down-regulating Wnt/ β-catenin signal transduction. Kansara et al. found that targeted knockout mouse WIF-1 could promote radiation-induced osteosarcoma, while silencing WIF-1 with hypermethylation promoter in primary human osteosarcoma was associated with increased β-catenin, cell proliferation and decreased differentiation. These data suggest that the elimination of Wnt antagonists can eliminate the inhibition of Wnt signal, resulting in an increase in the risk of osteosarcoma. In our research, we found that Hic-5 could interact with smad4 and inhibit activate of β-catenin.In summary, we revealed that the mechanism of Hic-5 regulated osteosarcoma development via inhibiting Wnt/β-catenin signal pathway.

## CONCLUSION

In conclusion, silencing Hic-5 prevented proliferation and promoted apoptosis of osteosarcoma through inhibiting Wnt/β-catenin signal via exosome pathway.

## MATERIALS AND METHODS

### Clinical samples

Fresh tissue samples and adjacent normal tissue samples were collected from 15 osteosarcoma patients at People’s Hospital of Rizhao (data shown in Table 1). This experiment has got the approval of the Medical Ethics Committee of People’s Hospital of Rizhao and this study is in line with the Declaration of Helsinki.

**Table 1:** Clinical features.

**Features**	**Osteosarcoma(N)**	**Percentage**	**Normal bone tissue(N)^+^**	**Percentage**
**Age(year)**				
<12	1	6.7%	1	6.7%
13-20	4	26.7%	4	26.7%
21-30	5	33.3%	5	33.3%
31-40	3	20.0%	3	20.0%
41-	2	13.3%	2	13.3%
**Sex**				
Male	9	60%	9	60%
Female	6	40%	6	40%
**Localization**				
Distal femur	1	6.7%	1	6.7%
Proximal tibia	4	26.7%	4	26.7%
Humerus	3	20.0%	3	20.0%
Tibia diaphysis	5	33.3%	5	33.3%
Other	2	13.3%	2	13.3%

### Cell culture

MG-63, HOS, U2-OS, SAOS-2 and hFOB1.19 cell lines were purchased from the Science Cell Laboratory. Cells were cultured in MEM (GIBCO, USA) supplemented with 10 % fetal bovine serum (Cromwell, USA) and 100 μL/mL penicillin and streptomycin (Sigma-Aldrich, USA) and placed at 37°C with 5% CO2.

### Cell transfection

RFP-shRNA was produced by Genomeditech, Co. (Shanghai, China). A scrambled shRNA was used as the negative control (sh-NC). About 5×10^5^ cells per well were seeded in 6 well plates, transfection of siRNA into the cells was performed using lipo2000 (Thermo Fisher Scientific, USA) according to the manufacturer’s recommendations. Cells were transfected with 20 nmol/L siRNA for 48 h, and then siRNA transfection in the best condition was performed.

### Real time-PCR

Total RNA was isolated from cells and tissues according to a standard protocol. And then, the purity and concentration of RNA was detected and all the samples were converted into cDNA using reverse transcription kit. We used SYBR Green (Thermo Fisher Scientific) system to perform the qRT-PCR.

### Western blot

Total protein was collected from cells with RIPA lysis Mix. Briefly, 50 μg protein extraction was loaded *via* SDS-PAGE and transferred onto nitrocellulose membranes (Life Sciences, Mexico), the membranes were incubated in 5% non-fat milk blocking buffer for 2 h. Then incubated with primary antibodies for 2 h at room temperature, then plated at 4 °C overnight, After incubation with secondary antibodies, the membranes were readied using an Odyssey, and data were analyzed with Odyssey software (LI-COR, USA). The primary antibody brand and usage concentration were as follow. Hic-5 (proteintech, 10565-1-AP, 1:500), Bcl2 (proteintech, 12789-1-AP, 1:500), Bax (proteintech, 60267-1-Ig, 1:500), Cleaved-caspase3 (CST, Asp175 1:1000), CD63 (proteintech, 25682-1-AP, 1:500), Alix (proteintech, 12422-1-AP, 1:500), Tsg101 (proteintech,14497-1-AP, 1:500), GM130 (proteintech,11308-1-AP, 1:500), smad4 (proteintech, 10231-1-AP, 1:500), sFRP1 (proteintech, 26460-1-AP, 1:500), wnt1 (proteintech, 27935-1-AP, 1:500), β-catenin (proteintech, 17565-1-AP, 1:500), and GAPDH (proteintech, 60004-1-Ig, 1:2000) was used as an internal control.

### Cell viability assay

MTT assay was used to assess cell viability. MG-63 and HOS cells were cultured in the 96-well plates for 1×10^3^/well. Overnight, cells were starved for 12 h. transfection reagent was added to the cells. Then cells were treated with 5 mg/mL MTT (20 μL/well) for 4 h. Removing supernatant, and adding DMSO (150 μL/well), the plate was shaking 30 min in room temperature. The absorbance was read at 490 nm using an Infinite 200PRO microplate spectrophotometer (BioTek, USA). The absorbance values were normalized to the control.

### *In vivo* tumor growth assay

Nude mice were purchased from Beijing Charles River. The nude mice were injected with treated-MG-63, MG-63 cells (5 × 10^6^) were subcutaneously injected in right lower limb of the nude mice. Tumor size was measured every 3 days. After another 15 d of injection, the tumor was removed for follow-up study.

### EDU assay

EdU incorporation. For EdU labeling, a 1:1,000 dilution of EdU-labeling reagent (Beyotime, China) was added to islet culture medium during the last 18 h of cell culture.

### Immunofluorescence staining

Cells were plated in a 24-well cell culture plate. The cells were washed by PBS and fixed with 4% paraformaldehyde, and permeabilized with 0.2% Triton-X-100 solution in PBS. Next, we blocked cell using goat serum. Then, the cells were incubated with Hic-5 antibody at 4 °C overnight followed with FITC-conjugated goat anti-mouse antibodies incubation for 1h. After three washes with PBS, we incubated cells by DAPI.

### Isolation and identification of exosome

When the cell fusion reached 70%, it was washed three times with 0.01mol/L phosphate buffer (PBS) and incubated with serum-free medium or exosome-free FBS medium for 48 hours. The supernatant was collected, centrifuged, 300xg centrifuged 10min at 4 °C to remove cells, 2000xg centrifugation 20min to remove cell fragments, and exudates were stored at -80 °C. Exosome morphology was observed by transmission electron microscope (TEM). The exosome particle size was detected by PSS Nicomp 380Z3000 analyzer (USA).

### Co-IP assay

Co-IP was performed in HEK 293T cells. The cells were collected and lysed in the whole cell extract buffer (400 mM KCl, 10 mM Na2HPO4, 1 mM EDTA, 1 mM dithiothreitol, 10% glycerol, 1 mM phenylmethylsulfonyl fluoride, 0.5% NP-40 with complete protease inhibitor cocktail). Appropriate antibodies and protein G Sepharose™ 4 fast flow beads were added to the extracts and mixtures were incubated overnight at 4 °C. After washing with the whole cell extract buffer, the beads were boiled in 1 × SDS-PAGE loading buffer (50 mM Tris-HCl, pH 6.8, 100 mM dithiothreitol, 2% SDS, 10% glycerol, 0.1% bromophenol blue). Samples were loaded and separated by SDS-PAGE and transferred to Immun-Blot™ PVDF membrane (Bio-Rad). The membrane was then incubated with appropriate primary antibodies (1:1000 dilution) followed by HRP-conjugated secondary antibodies (Beyontime, 1:2000 dilution). Protein signals were visualized by Western blotting detection reagents.

### Statistical analysis

All data is presented as a mean ± S.E.M. Statistical analysis was performed using Student's t-test or a one-way ANOVA.
